# Printed Thin Magnetic Films via Ternary Hybrid Diblock Copolymer Films Containing Magnetic Iron Oxide and Nickel Nanoparticles

**DOI:** 10.1021/acsami.4c18920

**Published:** 2024-12-11

**Authors:** Christopher R. Everett, Xinyu Jiang, Manuel A. Reus, Huaying Zhong, Martin Bitsch, Martina Plank, Markus Gallei, Matthias Opel, Matthias Schwartzkopf, Stephan V. Roth, Peter Müller-Buschbaum

**Affiliations:** † TUM School of Natural Sciences, Department of Physics, Chair for Functional Materials, Technical University of Munich, James-Franck-Street 1, 85748 Garching, Germany; ‡ Deutsches Elektronen-Synchrotron, Notkestraße 85, 22607 Hamburg, Germany; § Polymer Chemistry, Saarland University, Campus C4 2, 66123 Saarbrücken, Germany; ∥ Ernst-Berl-Institute for Technical and Macromolecular Chemistry, Technische Universität Darmstadt, Alarich-Weiss-Straße 4, 64287 Darmstadt, Germany; ⊥ Saarene, Saarland Center for Energy Materials and Sustainability, Campus C4 2, 66123 Saarbrücken, Germany; # Walther-Meissner-Institut, Bayerische Akademie der Wissenschaften, Walther-Meissner-Straße 8, 85748 Garching, Germany; ¶ Department of Fibre and Polymer Technology, KTH Royal Institute of Technology, Teknikringen 56-58, SE-100 44 Stockholm, Sweden

**Keywords:** ternary hybrid films, magnetic nanoparticles, printing, GISAXS, ferrimagnetic behavior

## Abstract

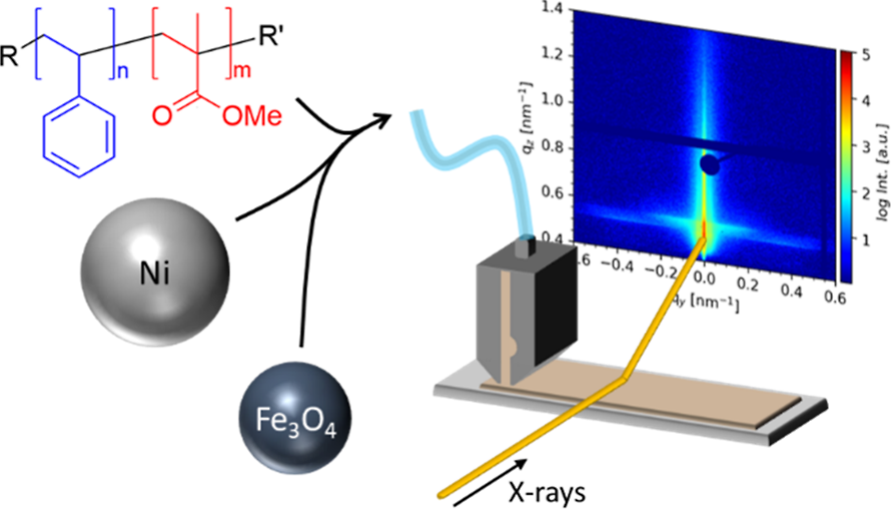

Ternary hybrid thin films composed of a diblock copolymer templating two types of nanoparticles (NPs) expand the functionality of binary systems, which renders them interesting for magnetic sensing or magnetic data storage applications. Herein, one-pot slot-die printed hybrid polystyrene-*block*-poly(methyl methacrylate) (PS-*b*-PMMA) thin films are prepared with iron oxide (magnetite, Fe_3_O_4_, *d* = 20 nm) and nickel NPs (Ni, *d* = 46 nm) in one step by the advanced slot-die coating technique, which facilitates upscaling of fabrication. The evolution of the hybrid film morphology is probed with in situ grazing-incidence small-angle X-ray scattering and compared to that of a PS-*b*-PMMA thin film without NPs. Additionally, scanning electron microscopy and atomic force microscopy are used to analyze the surface morphology of hybrid films with an increasing NP content after deposition. It is found that different from the pure PS-*b*-PMMA thin film drying kinetics with five stages, the ternary hybrid film formation can be divided into four stages that are attributed first to the wet film, solvent evaporation, a subsequent rapid coalescence and microphase separation, and finally the dry film. The magnetic properties of the hybrid thin films are investigated with a superconducting quantum interference device magnetometer. All hybrid films are ferrimagnetic and with increasing nickel weight percent in the hybrid film, while the iron oxide weight percent is kept constant, the magnetic properties of the film are modulated accordingly.

## 1 Introduction

Polymer-templated magnetic hybrid films can be deposited from solution by spin-coating or slot-die printing, the latter of which enables large-scale film fabrication under ambient conditions.^1,2^ In comparison, classical magnetic thin films are often fabricated utilizing a physical vapor deposition process such as sputtering, which can be limited by slow deposition rates.^3,4^ Therefore, wet chemical processing of thin magnetic films is of high interest as an alternative fabrication route for magnetic sensing or magnetic data storage applications. In this respect, the combination of a polymer film matrix with magnetic nanofillers is one common route for the wet chemical processing of thin magnetic films.

Diblock copolymers (DBCs) that undergo microphase separation to form thin films with periodic, ordered nanostructures are interesting in such scenarios to be the host matrix.^5−10^ By tuning the volume fraction of the amphiphilic blocks, a variety of nanostructures are accessible, including spheres, cylinders, and lamellae.^11,12^ In addition, with a constant interaction parameter, χ, the size of the nanostructured domains depends on the molecular weight of the polymer chain. By utilizing high or ultrahigh molecular weight (UHMW) DBCs, *M*_n_ > 5 × 10^5^ g mol^–1^, domain sizes of *d* > 80 nm can be achieved.^13−18^ Such large domains in DBC films are attractive for use as templates and scaffolds for inorganic materials such as magnetic nanoparticles (NPs). In particular, larger-size NPs can be accommodated by the individual domains of the DBC without risking a morphology perturbation upon NP addition.^19^

The arrangement of NPs inside DBC films is determined in part by the NP size and surface functionality. NPs with no surface functionality, i.e., nonselective for both of the polymer domains, localize either to the interface between the two blocks if the NPs are small or are expelled from the film due to entropy loss if the NPs are too large.^20−22^ Through surface functionalization, more precise NP localization can be achieved, and a specific domain of the DBC film can be targeted.^23,24^ Among the multitude of magnetic NPs, magnetite (Fe_3_O_4_) NPs are well-studied ferrimagnetic NPs that show size-dependent magnetic properties. In particular, at a critical size attributed to the transition from a single magnetic domain to multiple magnetic domains, a maximum in the coercivity (*H*_c_) is observed.^25^ However, this critical size is highly dependent on the crystal structure and by extension on the synthesis method.^25−27^ Nickel is a well-known soft ferromagnet with high permeability.^28^ Similar to Fe_3_O_4_ NPs, Ni NPs can demonstrate size-dependent magnetic behavior that is influenced by crystal size and synthesis method.^29−32^ Fe_3_O_4_ and Ni composites have shown interesting magnetic anisotropy attributed to the coupling between the magnetic domains.^28^ Thus, it is of interest to examine the magnetic behavior of Fe_3_O_4_ NPs and Ni NPs templated by a DBC.

Previous studies on hybrid DBC/NP thin films have focused on the one-step deposition of binary DBC/NP composites, i.e., solvent mixtures of one DBC and one type of NP deposited at the same time.^19,33−37^ In order to have more control over the properties of hybrid thin films, there is an interest in expanding on the development of ternary hybrid DBC/NP thin films with two types of NPs.^38−42^ In the case of nonmagnetic NPs, Bockstaller et al. used a one-step deposition approach for ternary composites by utilizing polystyrene-*block*-poly(ethylene propylene) (PS-*b*-PEP) and aliphatic-coated silica and gold NPs. While both particles were functionalized to be specific for the PEP block, the small gold NPs (*d* = 3.5 nm) were found to preferentially segregate to the PS/PEP interface, while the larger silica NPs (*d* = 21.5 nm) were found in the middle of the PEP domains.^41^ Horechyy et al. demonstrated a two-step approach for the dual patterning of NPs on a polystyrene-*block*-polyvinylpyridine (PS-*b*-PVP) template. After deposition of PS-*b*-PVP and Ag NPs (*d* = 12 nm) from a common solution to form binary thin films, the films were immersed in an aqueous dispersion of a second type of NP (*d* ≈ 2–5 nm), and an ordered ternary film was realized.^42^ However, ternary thin films containing two types of magnetic NPs of large size (*d* ≥ 20 nm) have not been the focus of previous research. Actually, such ternary magnetic thin films are interesting for magnetic sensing or magnetic data storage applications due to the better tunability of the magnetic properties.

In this work, ternary thin films with varying NP concentrations are prepared in a one-step deposition process by slot-die printing. In order to achieve domain sizes large enough to accommodate the large NPs, UHMW PS-*b*-PMMA is chosen. The morphology evolution of the ternary hybrid thin films containing PS-*b*-PMMA, Fe_3_O_4_ NPs, and Ni NPs is investigated by in situ grazing-incidence small-angle X-ray scattering (GISAXS) during slot-die printing, which probes the formation of interfacial nanostructures with high sample statistics. Atomic force microscopy (AFM) and scanning electron microscopy (SEM) are used to examine the local surface properties of the prepared films. Using a superconducting quantum interference device (SQUID) magnetometer, we probed the magnetic properties of the ternary films at two different temperatures. This analysis of thin film formation kinetics and the resulting magnetic properties of ternary DBC/NP/NP composites provides insight for the further design and fabrication of functional hybrid magnetic thin films with an increased complexity as compared with the previously studied binary DBC/NP composites. Such an increase in the complexity of the magnetic response is required to step forward in the area of printed magnetic thin films for magnetic sensing or magnetic data storage applications, while achieving printed magnetic bit-patterned media will be even more demanding.

## 2 Experimental Section

### 2.1 Materials

An asymmetric polystyrene-*block*-poly(methyl methacrylate) (PS-*b*-PMMA) DBC was synthesized by sequential anionic polymerization as described in a previous publication.^43^ The synthesized PS-*b*-PMMA had an average molar mass (*M*_n_) of 1307 kg mol^–1^ with a polydispersity index (*D̵*) of 1.18 and a PMMA volume fraction (φ_PMMA_) of 20.3%. Iron oxide NPs (Fe_3_O_4_, *d* = 20 ± 2.5 nm) coated in oleic acid ligands and suspended in toluene at a concentration of 5 mg mL^–1^ were purchased from Sigma-Aldrich. Nickel NPs (Ni, *d*_TEM_ = 46 ± 10 nm, Figures S1 and S2) were synthesized by a chemical precipitation route according to Chopra et al.^44^ The NPs were then functionalized with PMMA ligands. Details of the synthesis can be found in the Supporting Information. The NPs were suspended in toluene at a concentration of 5 mg mL^–1^.

### 2.2 Preparation of PS-*b*-PMMA/Nanoparticle Films

PS-*b*-PMMA was dissolved in toluene (Sigma-Aldrich) at a concentration of 10 mg mL^–1^ and shaken overnight at room temperature. Various concentrations of Fe_3_O_4_ NPs and Ni NPs were added to the polymer solution. For the current investigation, four different weight ratios (wt %/wt %) of Fe_3_O_4_ NPs to Ni NPs (no NPs, 1:1, 1:2, and 1:5), with respect to PS-*b*-PMMA, were selected. The NPs were added to the polymer solution 1 h before the in situ GISAXS investigation. Precleaned Si substrates (p/Bor, ⟨100⟩, *d* = 525 ± 25 μm, Si-Mat) cut to 2.5 cm × 6 cm were prepared using an acid solution, as detailed in the Supporting Information. The Si substrates were placed on the stage of a custom-built meniscus-guided slot-die printer.^45^ Above the stage, a syringe pump pushed the polymer/NP solution into an aluminum print head. After the formation of a stable meniscus, the print head was moved across the Si substrate perpendicular to the X-ray beam to deposit the film. The printing parameters for film deposition and formation were determined through pretests carried out in the laboratory at TUM. The gap height between the print head and substrate was 6 mm, while the printing speed was 5 mm s^–1^. The acceleration of the print head from rest to final printing speed was 10 mm s^–2^. During the printing process, the pump rate of the solution into the print head was set to 0.2 mL min^–1^. The printed range of the slot-die coating process was 5.2 cm. Film deposition and formation were performed at room temperature. With the above-mentioned printing procedure, further tests for reproducibility were performed. In total, 6 films were prepared for each system to confirm the reproducibility of the printing procedure.

### 2.3 In Situ Characterization

Using the printing procedure described in Section 2.2, the evolution of the films’ morphology during self-assembly was followed with in situ GISAXS measurements performed at the MiNaXS/P03 beamline of PETRA III at DESY in Hamburg, Germany.^46^ For each weight ratio of NPs (no NPs, 1:1, 1:2, and 1:5), one film was investigated in situ. The wavelength of the X-ray beam was 0.1048 nm, and an incidence angle of 0.4°, above the critical angle of the DBC and NPs, was selected. The sample–detector distance was 4122 mm. A two-dimensional detector (Pilatus 2M, Dectris Ltd.) with a pixel size of 172 μm × 172 μm was used to record the scattered signal. To avoid overexposure of the hybrid film, an exposure time of 0.1 s was chosen and a fresh position was measured for each captured image. The thickness of each hybrid film was determined to be approximately 300 nm (Table S1). The in situ printed hybrid films were further investigated with the ex situ characterization techniques.

### 2.4 Ex Situ Characterization Techniques

The surface morphology of the hybrid films after deposition was investigated with AFM (CoreAFM, Nanosurf) operated in tapping mode in air utilizing a monolithic silicon cantilever coated with aluminum (TAP190Al-G, BudgetSensors) and SEM (Gemini Nvision 40, ZEISS). The magnetic behavior of the hybrid films was investigated with a SQUID magnetometer (MPMS XL-7, Quantum Design) in direct current mode. The magnetic moment was measured in the film plane and an external magnetic field (−70,000 to 70,000 Oe) was applied parallel to the film surface. The magnetic properties were probed at 300 K and 40 K.

## 3 Results and Discussion

### 3.1 As-Printed Surface Morphology

**Figure 1:**
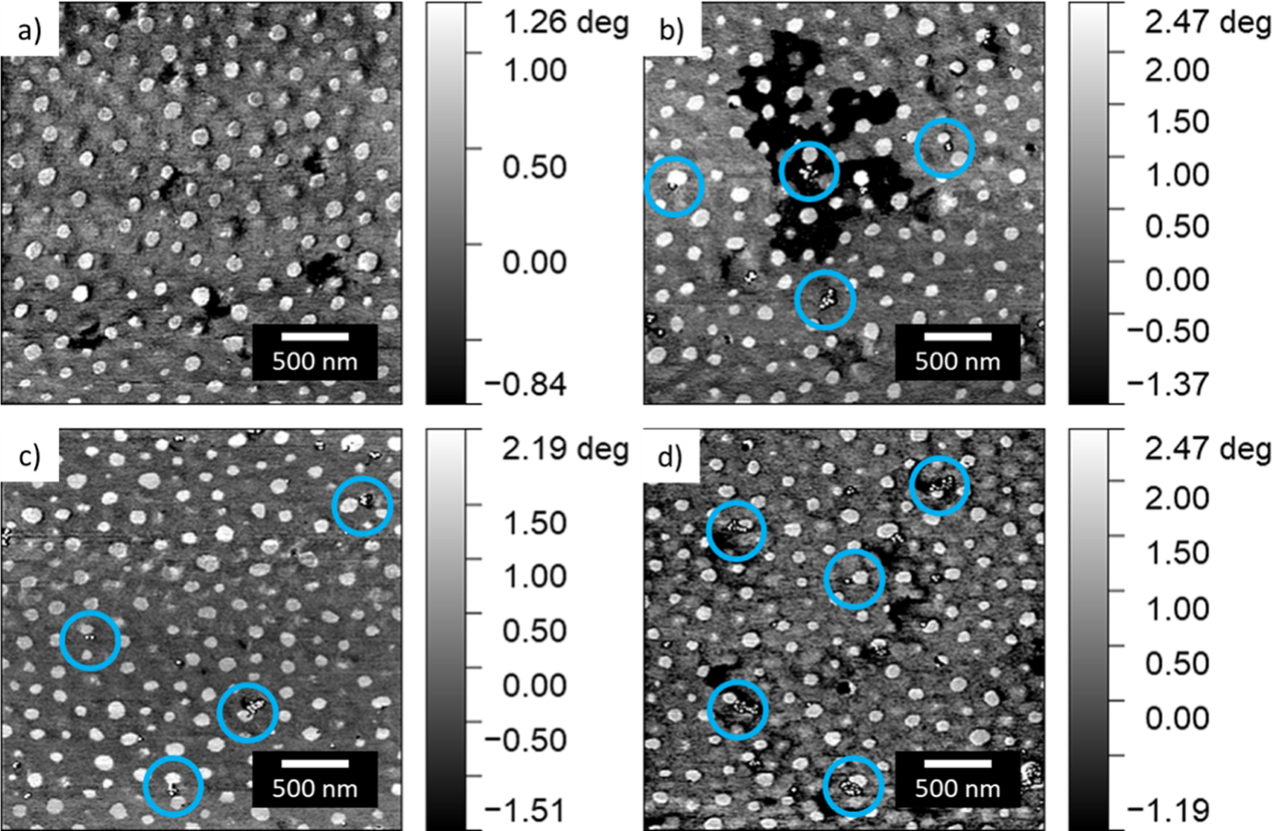
AFM phase images of ternary hybrid films printed during the in situ characterization with increasing NP concentration: (a) no NPs, (b) 1 wt % Fe_3_O_4_ and 1 wt % Ni, (c) 1 wt % Fe_3_O_4_ and 2 wt % Ni, (d) 1 wt % Fe_3_O_4_ and 5 wt % Ni. The bright domains correspond to the PMMA block while the darker matrix corresponds to PS. NPs appear individually or as bright, small aggregates typically found at the domain interface (blue circles).

The surface morphology of the as-printed films is investigated on the microscale and nanoscale by complementary AFM and SEM measurements. Due to the utilization of a DBC of UHMW, the segregation strength (χ*N*) between the PS blocks and the PMMA is large, where *N* is the total degree of polymerization. Accordingly, for large segregation strengths, the self-consistent mean-field theory predicts the formation of cylindrical PMMA domains inside the thin film when the PMMA volume fraction is 0.2.^47,48^ This agrees with the observations made in our previous study.^43^ The AFM phase image of the as-printed pure PS-*b*-PMMA film can be seen in Figure 1a and clearly reveals the DBC morphology. The PMMA domains appear as bright circular spots in the darker PS matrix due to the relative viscoelastic contrast between the blocks.^49,50^ As the concentration of NPs increases, the embedded NPs appear individually or as small aggregates on the film surface (blue circles). Corresponding AFM topography images are shown in Figure S3. The bright circular spots corresponding to an increased height are assigned to the PMMA domains, while the darker area in between is assigned to the PS matrix. Toluene is a good solvent for both PS and PMMA but has a higher affinity to PS.^51^ During drying, as the PS selective solvent leaves the film, the PMMA domains become glassy while the PS domains remain more mobile, leading to PMMA protrusions in the dry, as-casted thin film.^52^ Due to the film thickness not matching the DBC periodicity, holes, which are not deep enough to expose the bare substrate underneath, appear in the film.^53^

As the two types of NPs are coated with different surface ligands, oleic acid for the Fe_3_O_4_ NPs and PMMA groups for the Ni NPs, segregation of the NPs into a specific domain, Fe_3_O_4_ NPs into PS and Ni NPs in PMMA, should be energetically favorable. However, the individual NPs and small NP clusters localize at the interface of PS and PMMA or appear to sit on the surface of the film. This is due in part to the high segregation strength, χ*N*, of the UHMW DBC, where an increase in χ*N* leads to an increase in the interfacial tension between the two blocks. Thus, even though the NPs are selective, a reduction in the interfacial tension is more energetically favorable and the NPs localize to the polymer interface.^21^

**Figure 2:**
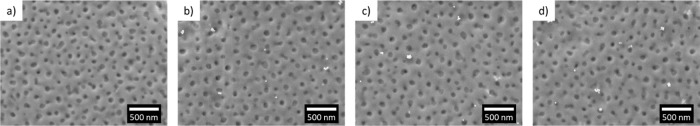
SEM images of ternary hybrid films printed during the in situ characterization with increasing NP concentration: (a) no NPs, (b) 1 wt % Fe_3_O_4_ and 1 wt % Ni, (c) 1 wt % Fe_3_O_4_ and 2 wt % Ni, (d) 1 wt % Fe_3_O_4_ and 5 wt % Ni. The NPs, appearing as white dots, appear as clusters or as individual NPs on/in the thin films.

Corresponding SEM measurements are also carried out on the as-printed films. The SEM image of the as-printed pure PS-*b*-PMMA film can be seen in Figure 2a. The bright matrix is PS, while the dark domains are PMMA, resulting from the compositional contrast between the two blocks that depends on the effective atomic number, *Z*_eff_.^54,55^ At low photon energies near 2 keV, *Z*_eff_ of PS is higher than that of PMMA. Thus, the PS matrix appears brighter in the SEM images than the PMMA domains, as opposed to the phase and topographical information obtained from the AFM analysis. In addition to the circular PMMA domains, elongated domains are also observed. As the self-assembly process occurs over a short time period, the reorganization of the polymer domains is cut short, and the film remains in a nonequilibrium state. Furthermore, a few inorganic NPs are readily visible on the surface of the hybrid films, and the NPs appear individually or as clusters at or near the interface between the two polymer domains.

### 3.2 In Situ Morphology Evolution

The influence of the NPs on the self-assembly of the DBC films is studied during slot-die printing by in situ GISAXS. The grazing-incidence geometry provides information on the film morphology with high statistical significance.^56,57^ Printing is done at ambient temperature to observe the transition from the dispersion/wet-film regime to the dry thin film. As the print head moves across the substrate and through the incoming beam path, *t* = 0 s is defined as the first scattering image where the signal from the deposited dispersion is observed.

Selected 2D GISAXS data showing the evolution of the scattering information during film formation are shown in Figure S4 in the Supporting Information for the PS-*b*-PMMA film with no NPs. Initially, only scattering from the solution is observed, and no information relating to the DBC film morphology can be resolved. As self-assembly begins and the film transitions from a wet to a dry state, lateral features in the scattering information develop, which correspond to the DBC domains.

**Figure 3:**
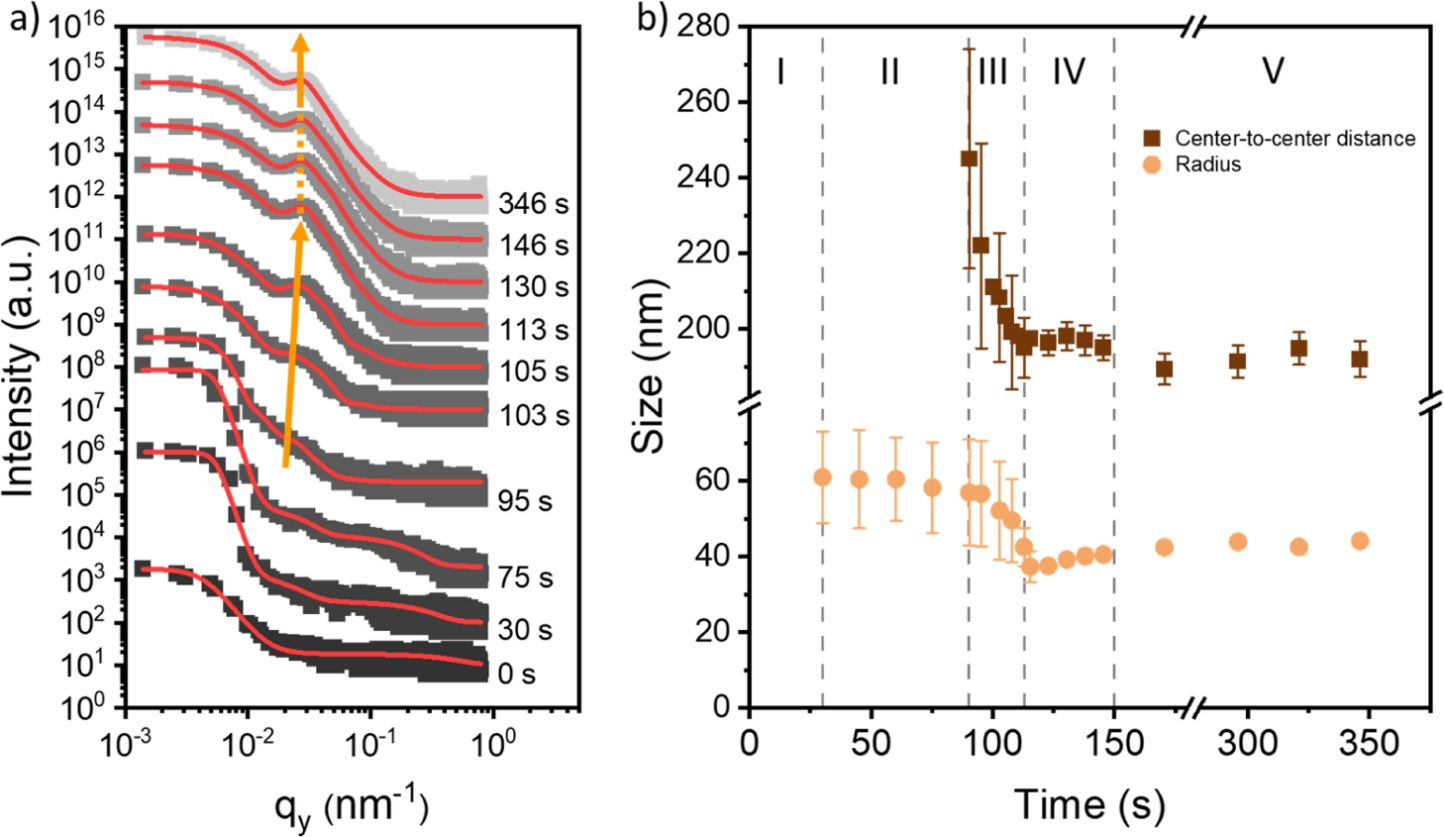
(a) Representative line cuts are taken from the 2D GISAXS data of the PS-*b*-PMMA film printed during the in situ characterization containing no NPs. The curves are shifted along the *y*-axis for clarity, and the time at which each curve is collected during the printing process is displayed to the right of each curve. Fits for the data are shown in red and overlaid on the respective curve. As the printing process proceeds, the evolution of the polymer domain peak can be clearly observed (orange arrow). (b) Extracted radius and distance information for the cylindrical PMMA domains obtained from the fits. The film formation process can be divided into five stages (I–V): wet film, solvent evaporation, coalescence and microphase separation, morphology relaxation, and dry film.

To quantify the changes observed in the scattering information relating to the DBC morphology, horizontal line cuts at the Yoneda region of the two blocks, which have similar critical angles (α_c,PS_ ≈ 0.10°, α_c,PMMA_ ≈ 0.11°), are taken from the 2D GISAXS data.^56^ Exemplary line cuts are shown in Figure 3a with the corresponding fits overlaid in red. The line cuts show one main feature that is attributed to the PMMA domains. The data are modeled according to the distorted-wave Born approximation and the effective interface approximation.^57−59^ The extracted radii and center-to-center distances from the model are plotted in Figure 3b.

The formation of the DBC film without NPs can be divided into five stages based on the drying kinetics. A 2D intensity mapping of the horizontal line cuts with the ascribed stages can be seen in the Supporting Information (Figure S5). The first stage (0 < *t* < 30 s) is characterized by the wet film. For the first 30 s after deposition, no significant scattering features are observed from the wet film so that a preaggregation in the solution is ruled out. In the second stage (30 < *t* < 95 s), due to solvent evaporation, a shoulder-like scattering feature evolves located near *q*_y_ = 0.02 nm^–1^, which is attributed to the form factor of the PMMA domains, i.e., the radius, with an average size of (61 ± 12) nm. The domains remain unordered in relation to one another, so that no clear structure factor related peak is seen. A broad peak (q_*y*_ ≈ 0.1–0.5 nm^–1^) of low intensity observed in both the first and second stages is ascribed to background scattering from the wet film. In the third stage (95 < *t* < 113 s), the shoulder-like scattering feature changes into a peak (marked with an orange arrow in Figure 3a) due to the onset of microphase separation. This polymer domain peak shifts toward higher *q*_y_ values, which is accompanied by an increase in intensity. Interdomain ordering can be observed, and at the beginning of this stage, the center-to-center distance is (250 ± 30) nm. Over a period of approximately 20 s, the size and center-to-center distance of the PMMA domains decrease markedly to (43 ± 5) nm and (195 ± 8) nm. A rapid coalescence and microphase separation in the DBC film occurs in conjunction with ongoing solvent evaporation. The fourth stage (113 < *t* < 150 s) describes the slow transformation of the film into the dry state with little changes in intensity and domain peak position. Within this time frame, the center-to-center distance is consistently larger, and the domain radius is consistently smaller than the values attributed to the dry film in the following stage. Since only a little residual solvent evaporates from the film, only subtle changes in the film morphology are observed as the polymer chains relax toward a more favorable chain conformation.^60,61^ In the dry film (*t* > 150 s), due to the absence of chain mobility, a final morphology results with a constant radius of (44 ± 2) nm and a constant center-to-center distance of (192 ± 5) nm.

**Figure 4:**
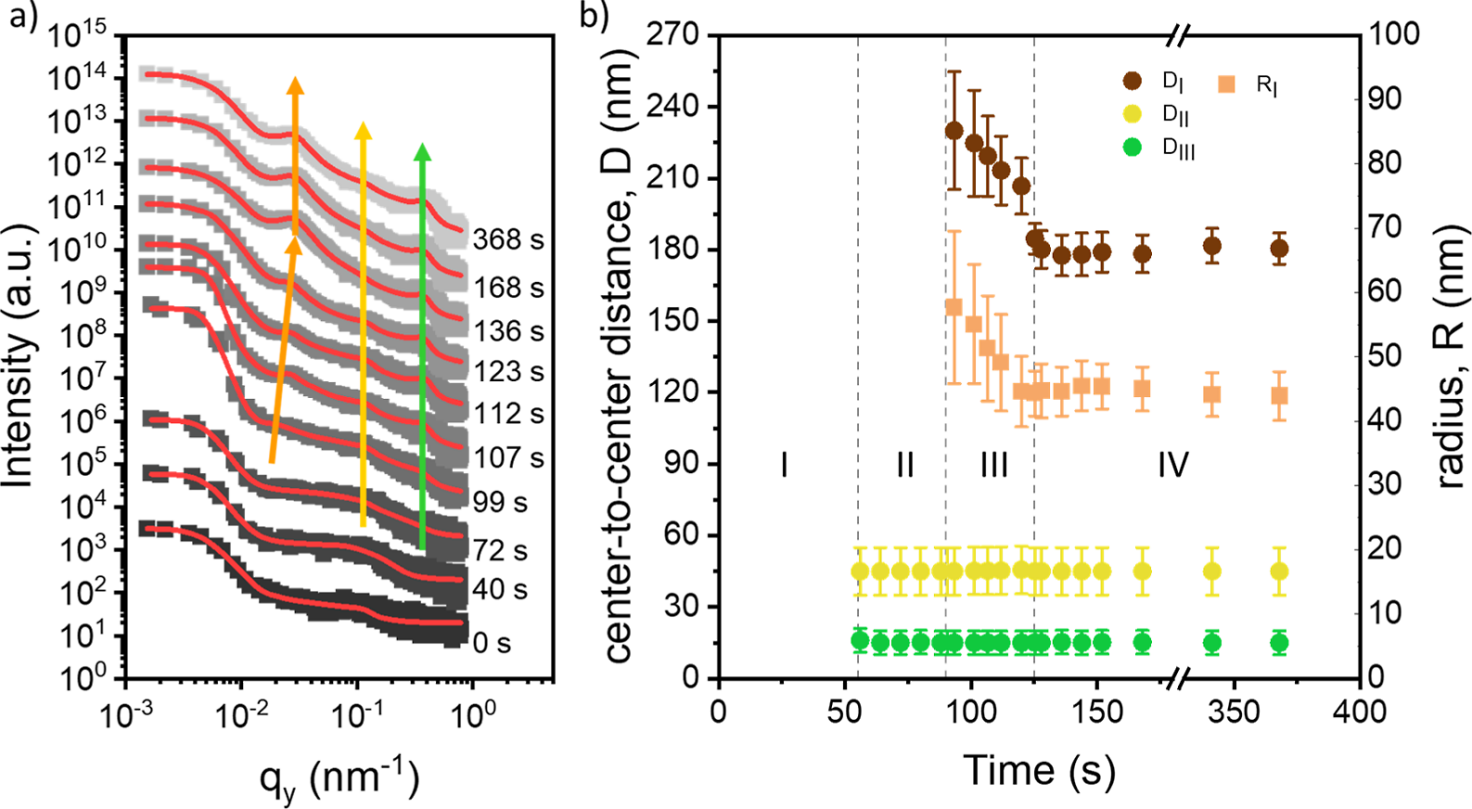
(a) Representative line cuts taken from the 2D GISAXS data of the PS-*b*-PMMA film printed during the in situ characterization containing 1 wt % Fe_3_O_4_ NPs and 1 wt % Ni NPs. The curves are shifted along the *y*-axis for clarity and the time at which each curve is collected during the printing process is displayed to the right of each curve. Fits for the data are shown in red and overlaid on the respective curve. As the printing process proceeds, the evolution of the polymer domain peak can be clearly observed (orange arrow). Peaks corresponding to the Ni NPs and Fe_3_O_4_ NPs are highlighted with yellow and green arrows, respectively. (b) Extracted radius and distance information for the cylindrical PMMA domains (D_I_ and R_I_), Ni aggregates (D_II_), and Fe_3_O_4_ aggregates (D_III_) obtained from the fits. The film formation process can be divided into four stages (I–IV): wet film, solvent evaporation, coalescence and microphase separation, and dry film.

For the in situ GISAXS study of the printing of the ternary hybrid DBC film, we restrict ourselves to the example containing 1 wt % Fe_3_O_4_ NPs and 1 wt % Ni NPs. The horizontal line cuts and extracted morphological information are shown in Figure 4a,b, in addition to selected 2D GISAXS data shown in Figure S6. Compared with the pure DBC film, the hybrid film with mixed magnetic NPs shows only four stages of film formation, consistent with the 2D intensity map of the horizontal line cuts shown in Figure S7. The first stage (0 < *t* < 55 s) corresponds to the wet film, and no scattering features are seen. Thus, no characteristic morphological feature developed as in the pure DBC. In a further similarity to the pure DBC film, a broad scattering feature around 0.1 to 0.5 nm^–1^ is observed. In the second stage (55 < *t* < 90 s), during solvent evaporation, two small peaks at high *q*_y_ values, *q*_y_ = 0.18 nm^–1^ and *q*_y_ = 0.36 nm^–1^, appear in the GISAXS data. These peaks correspond to the Ni NPs and Fe_3_O_4_ NPs (yellow and green arrows in Figure 4a), which are modeled as aggregates in which the structure factor is twice the form factor. The beginning of the third stage (90 s < *t* < 125 s) is defined by the abrupt appearance of the polymer domain peak near *q*_y_ = 0.019 nm^–1^, and the PMMA domains have an average radius of (58 ± 12) nm and center-to-center distance of (230 ± 30) nm. Over the course of the third stage, solvent evaporation and coalescence, the polymer domain peak increases in intensity, and the peak moves to higher *q*_y_ values. This is reflected in the decrease in the average radius and center-to-center distance to (44 ± 6) nm and (185 ± 6) nm. No changes in the NP peak positions are observed as the NPs remain as aggregates and the individual NP size does not change. The fourth and final stage (*t* > 125 s) represents the dry film with a constant PMMA domain radius of (44 ± 4) nm and center-to-center distance of (181 ± 7) nm. These values are similar to those of the pure DBC film, suggesting that the film morphology, even in the presence of NP aggregates, is undisturbed at such low NP concentrations. The absence of an observable relaxation in the polymer can be attributed to heavy and sterically demanding NPs and their agglomerations in the hybrid film. As discussed in the analysis of the SEM images, the NPs preferentially localized to the interface between the PS and PMMA domains in order to reduce the interfacial tension between the two blocks. Therefore, the further reorganization of the polymer domains during residual solvent evaporation is energetically unfavorable.

The rates of self-assembly of the pure DBC film and the hybrid film are compared by examining the time it takes for coalescence and microphase separation to occur (stage III for both films). Compared to the pure DBC film, where self-assembly occurred rapidly in approximately 18 s, the hybrid film containing 1 wt % Fe_3_O_4_ NPs and 1 wt % Ni NPs undergoes a slower self-assembly process in approximately 35 s. This behavior is attributed to the large size of the NPs and the presence of NP agglomerates in the film, which sterically hinder the organization of the DBC morphology and slow down the self-assembly process.

The distribution of the NPs throughout the bulk of the DBC films is confirmed by analyzing the vertical line cuts of the 2D GISAXS data of the as-prepared thin films. The vertical cuts of the pure DBC film and two hybrid films containing increasing NP concentrations can be seen in Figure S8 in the Supporting Information. The positions of the PS and PMMA Yoneda peaks appear in the pure DBC at in-plane scattering angles of φ = 0.501° for PS and φ = 0.510° for PMMA. These values correspond closely to the sum of the incidence angle (α_*i*_ = 0.4°) and the respective critical angles of PS (α_c_ = 0.102°) and PMMA (α_c_ = 0.111°) calculated for the X-ray wavelength used in this investigation. Upon the addition of 1 wt % Fe_3_O_4_ NPs and 1 wt % Ni NPs, the value of the Yoneda peaks for both PS and PMMA shifts to higher angles. This results from the increase in the electron density of the PS matrix and of the PMMA cylindrical domains as the NPs localize inside the respective domains in the films. Upon further increase of the Ni NP concentration to 5 wt %, the critical angle of PMMA further increases while the critical angle of PS remains constant as the concentration of Fe_3_O_4_ NPs is kept at 1 wt %. Therefore, while a relatively low number of NPs is observed on the surface of the hybrid films in the AFM and SEM analyses, the vertical line cuts confirm the successful embedding of the NPs within the films.

### 3.3 Concentration-Dependent Magnetic Properties

**Figure 5:**
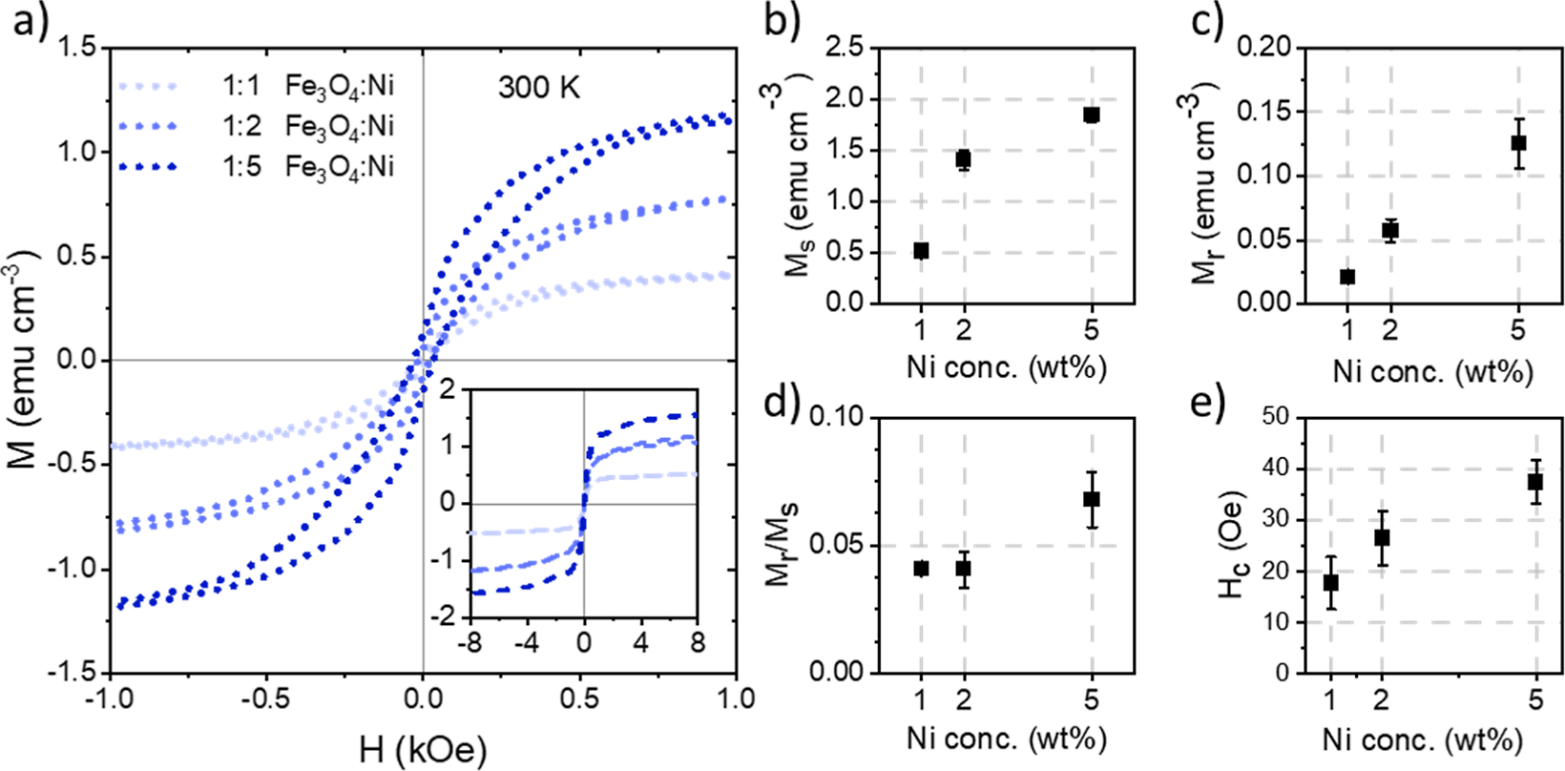
(a) Magnetic hysteresis curves for the ternary hybrid films printed during the in situ characterization measured at 300 K. For clarity, the curves are plotted between −1 kOe and 1 kOe. The inset shows the measured curves between −8 kOe and 8 kOe. (b) Saturation magnetization (*M*_s_), (c) remanence (*M*_r_), (d) squareness (*M*_r_/*M*_s_), and (e) coercivity (*H*_c_) extracted from the magnetization curves.

The concentration-dependent magnetic properties of the ternary hybrid films are investigated with a SQUID magnetometer. The Fe_3_O_4_ NPs are ferrimagnetic and Ni NPs are ferromagnetic.^25,31^ Typical magnetic hysteresis curves measured between −1 and 1 kOe at 300 K are observed, as shown in Figure 5a, and the films can be classified as relatively soft magnetic materials, i.e., low coercivity. The inset in Figure 5a shows the magnetization curves over a larger field range. The magnetization of the hybrid films is expressed in units M, which is given as the magnetic moment and is measured by the SQUID magnetometer per unit volume, calculated from the measured film dimensions. As expected, the saturation magnetization *M*_s_ of the hybrid films, plotted in Figure 5b, increases with increasing Ni content from 0.52 ± 0.01 emu cm^–3^ for the film containing 1 wt % Ni to 1.4 ± 0.1 emu cm^–3^ and 1.8 ± 0.1 emu cm^–3^ for the films containing 2 wt % Ni and 5 wt % Ni, respectively. The remanence *M*_r_ also increases linearly with respect to Ni concentration, Figure 5c, from 0.02 ± 0.01 emu cm^–3^ to 0.06 ± 0.01 emu cm^–3^ and 0.13 ± 0.02 emu cm^–3^. Due to the low remanence, the squareness ratio (*M*_r_/*M*_s_), Figure 5d, remains low for the hybrid films, around approximately 0.05. The higher the squareness value, the better suited the films are for potential magnetic data recording applications as a value close to unity indicates a stable device. In addition, a “bulge” can be observed in the hysteresis curves, which becomes pronounced with increasing Ni content. As the hysteresis curves of the ternary films are the superposition of Fe_3_O_4_ and Ni, this feature is described by the difference in field strength at which the magnetization of each NP type switches.^62,63^

While the concentration-dependent *M*_s_, *M*_r_, and squareness follow trends found in our previous work for single NP hybrid thin films, the coercivity *H*_c_ of the ternary films in this work demonstrates a unique behavior. With increasing Ni concentration, *H*_c_ also increases in a linear fashion. These values increase from 18 ± 5 Oe for the film containing 1 wt % Ni to 27 ± 5 Oe and 40 ± 4 Oe for the films containing 2 wt % Ni and 5 wt % Ni.

To better gain insight into the magnetic behavior of the ternary hybrid films, the magnetic properties of the films examined in this paper are compared to binary hybrid films containing either only 2 wt % Fe_3_O_4_ NPs or 2 wt % Ni NPs, as shown in Figure S9. In particular, when examining the *H*_c_, the binary film with 2 wt % Fe_3_O_4_ NPs shows a *H*_c_ value of about 71 Oe and the binary film with 2 wt % Ni NPs shows a value *H*_c_ of 2 Oe. All ternary hybrid films show *H*_c_ values above 2 Oe but below the value of 71 Oe associated with Fe_3_O_4_ NPs. This finding is attributed to the magnetic dipole interactions between the harder Fe_3_O_4_ NPs and the soft Ni NPs.^64−66^ Adding more Ni NPs into the ternary system may result in more Ni NP clusters, which might explain the increase in the coercivity with increasing Ni NP concentration.^66^

### 3.4 Temperature-Dependent Magnetic Properties

**Figure 6:**
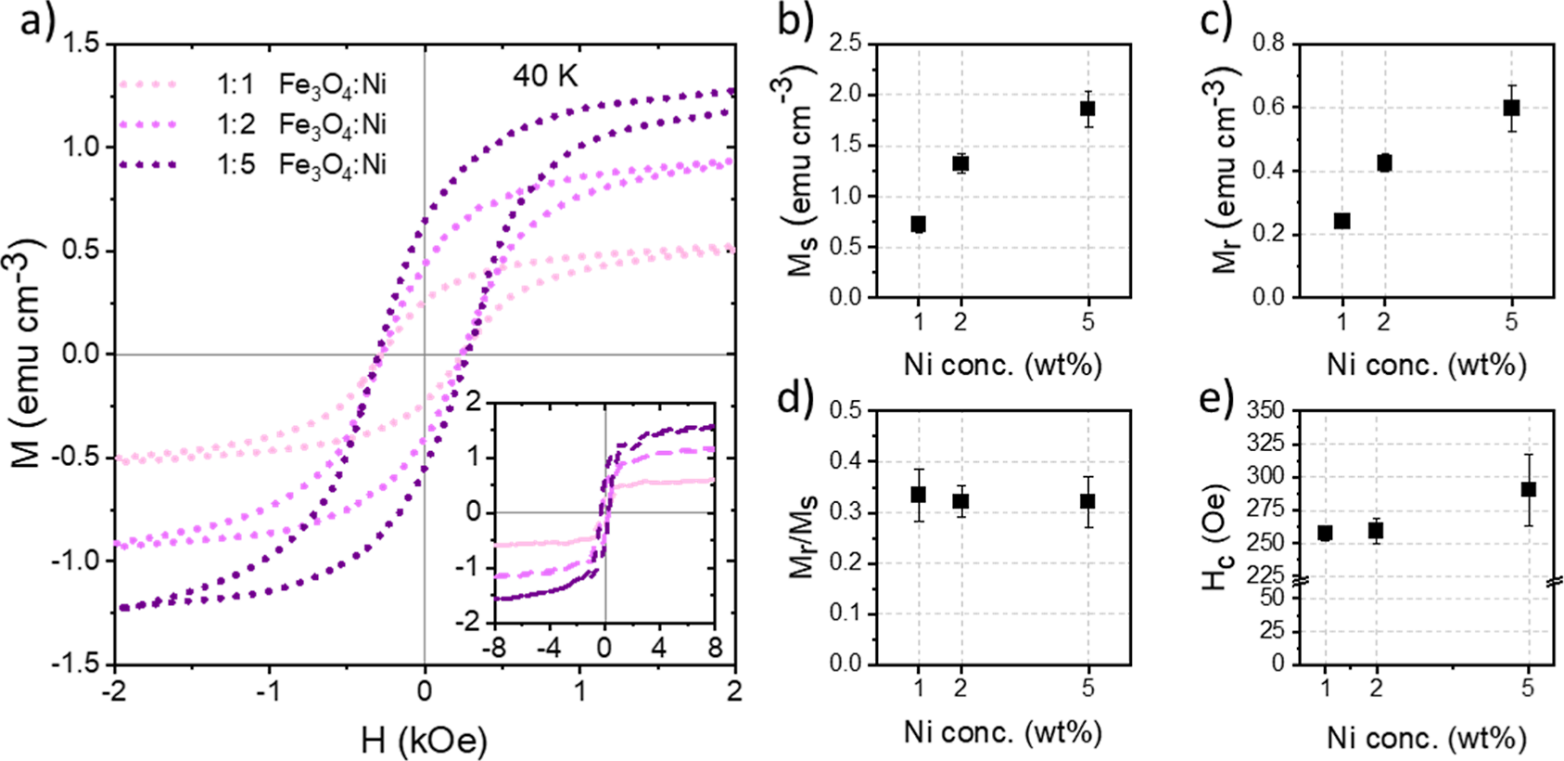
(a) Magnetic hysteresis curves for the ternary hybrid films printed during the in situ characterization measured at 40 K. For clarity, the curves are plotted between −2 kOe and 2 kOe. The inset shows the measured curves between −8 kOe and 8 kOe. (b) Saturation magnetization (*M*_s_), (c) remanence (*M*_r_), (d) squareness (*M*_r_/*M*_s_), and (e) coercivity (*H*_c_) extracted from the magnetization curves.

To determine the influence of temperature on the magnetic properties of the hybrid thin films, magnetic hysteresis curves for the ternary films measured between −2 and 2 kOe at 40 K are shown in Figure 6a. As expected, *M*_s_ remains unchanged. The inset in Figure 6a shows the magnetization curves over a larger field range. *M*_r_ and *H*_c_, however, show significant increases. This is due to the reduced thermal fluctuation and increased alignment of the magnetic domains. Therefore, while the number of magnetic domains does not change, which is related to the number of NPs, the magnetic field strength required to flip the domains increases.^67−69^ Here, the remanence has a value of 0.24 ± 0.02 emu cm^–3^ for the film containing 1 wt % Ni and increases approximately linearly to 0.43 ± 0.03 emu cm^–3^ and 0.60 ± 0.07 emu cm^–3^ for 2 wt % and 5 wt % Ni. Due to the increase in remanence, the squareness of the measured samples also increases and reaches a value of around 0.32 for all samples. The coercivity begins at a value of 257 ± 5 Oe for the 1 wt % Ni sample and increases to 259 ± 10 Oe and 290 ± 27 Oe for the 2 wt % Ni and 5 wt % Ni samples. Interestingly, the bulge in the hysteresis curves seen at 300 K disappears at 40 K. At this low temperature, the magnetization of both types of NPs switch at the same field strength and the film behaves like a single ferrimagnetic layer due to magnetic coupling.^62^

## 4 Conclusions

Ternary hybrid thin films composed of a DBC and two different types of magnetic NPs are fabricated in a one-pot slot-die coating process for magnetic sensing or magnetic data storage applications. GISAXS is used to track the film formation in situ and the morphology evolution of a reference PS-*b*-PMMA film with no NPs is compared to that of a PS-*b*-PMMA film containing both Fe_3_O_4_ and Ni NPs. The pure DBC film shows five stages of film formation and the ternary hybrid film shows four stages, illustrating the influence of NP addition on kinetics. First, in both cases, a wet film is observed after deposition. After a period of solvent evaporation in which the film begins to dry, a rapid coalescence and microphase separation occurs, which is accompanied by the appearance of the polymer domain scattering feature that further transforms into a well-defined peak in the scattering data. For the ternary hybrid thin film, the scattering information from the NP aggregates appears during the solvent evaporation stage and remains unchanged throughout the film formation process. The final stage is characterized by the dry film, where no changes in the film morphology happen due to restricted mobility. For the pure DBC film, an intermediate stage is observed before the beginning of the dry film stage, relating to the residual solvent remaining in the film, which enables a small polymer chain relaxation. Due to the large NP size and NP aggregation, the NPs inside the films are preferentially located in the polymer domains or at the PS/PMMA interface. In addition to the morphology investigation, the magnetic properties of the ternary films are examined. All films are ferrimagnetic, and the magnetic properties are modulated by tuning the NP concentration. Above all, the fabrication of functional ternary hybrid thin films in a one-pot slot-die synthesis method via printing demonstrates a simple method to modify the magnetic behavior of magnetic hybrid thin films for magnetic sensing or magnetic data storage applications while achieving printed magnetic bit-patterned media will require a better position control of the magnetic NPs.

## Supporting Information Available

Experimental details, synthesis of Ni NPs and corresponding XRD and TEM, AFM topography images, 2D GISAXS images and color maps, GISAXS modeling details, and magnetic properties of NPs (PDF)

## References

[ref1] Weller D. W.; Galuska L.; Wang W.; Ehlenburg D.; Hong K.; Gu X. Roll-to-Roll Scalable Production of Ordered Microdomains through Nonvolatile Additive Solvent Annealing of Block Copolymers. Macromolecules 2019, 52 (13), 5026–5032. 10.1021/acs.macromol.9b00772.

[ref2] Lee J.; Seo Y.-H.; Kwon S.-N.; Kim D.-H.; Jang S.; Jung H.; Lee Y.; Weerasinghe H.; Kim T.; Kim J. Y.; Vak D.; Na S.-I. Slot-Die and Roll-to-Roll Processed Single Junction Organic Photovoltaic Cells with the Highest Efficiency. Adv. Energy Mater. 2019, 9 (36), 1901805 10.1002/aenm.201901805.

[ref3] Miller E. L.; Paluselli D.; Marsen B.; Rocheleau R. E. Low-temperature reactively sputtered iron oxide for thin film devices. Thin Solid Films 2004, 466 (1–2), 307–313. 10.1016/j.tsf.2004.02.093.

[ref4] Manoj T.; Perumal H. P.; Paikaray B.; Haldar A.; Sinha J.; Bhattacharjee P. P.; Murapaka C. Perpendicular magnetic anisotropy in a sputter deposited nanocrystalline high entropy alloy thin film. J. Alloys Compd. 2023, 930, 167337 10.1016/j.jallcom.2022.167337.

[ref5] Song D.-P.; Li C.; Li W.; Watkins J. J. Block Copolymer Nanocomposites with High Refractive Index Contrast for One-Step Photonics. ACS Nano 2016, 10 (1), 1216–1223. 10.1021/acsnano.5b06525. 26713452

[ref6] Wei Q.; Lin Y.; Anderson E. R.; Briseno A. L.; Gido S. P.; Watkins J. J. Additive-driven assembly of block copolymer-nanoparticle hybrid materials for solution processable floating gate memory. ACS Nano 2012, 6 (2), 1188–1194. 10.1021/nn203847r. 22272941

[ref7] Eren N.; Burg O.; Michman E.; Popov I.; Shenhar R. Gold nanoparticle arrays organized in mixed patterns through directed self-assembly of ultrathin block copolymer films on topographic substrates. Polymer 2022, 245, 124727 10.1016/j.polymer.2022.124727.

[ref8] Pula P.; Leniart A. A.; Krol J.; Gorzkowski M. T.; Suster M. C.; Wrobel P.; Lewera A.; Majewski P. W. Block Copolymer-Templated, Single-Step Synthesis of Transition Metal Oxide Nanostructures for Sensing Applications. ACS Appl. Mater. Interfaces 2023, 15 (50), 57970–57980. 10.1021/acsami.3c10439. 37644616 PMC10739603

[ref9] Yabu H.; Matsui J.; Hara M.; Nagano S.; Matsuo Y.; Nagao Y. Proton Conductivities of Lamellae-Forming Bioinspired Block Copolymer Thin Films Containing Silver Nanoparticles. Langmuir 2016, 32 (37), 9484–9491. 10.1021/acs.langmuir.6b02521. 27589224

[ref10] Upadhyaya L.; Semsarilar M.; Fernández-Pacheco R.; Martinez G.; Mallada R.; Coelhoso I. M.; Portugal C. A. M.; Crespo J. G.; Deratani A.; Quemener D. Nano-structured magneto-responsive membranes from block copolymers and iron oxide nanoparticles. Polym. Chem. 2017, 8 (3), 605–614. 10.1039/C6PY01870J.

[ref11] Mai Y.; Eisenberg A. Self-assembly of block copolymers. Chem. Soc. Rev. 2012, 41 (18), 5969–5985. 10.1039/c2cs35115c. 22776960

[ref12] Huang C.; Zhu Y.; Man X. Block copolymer thin films. Phys. Rep. 2021, 932, 1–36. 10.1016/j.physrep.2021.07.005.

[ref13] Mapas J. K. D.; Thomay T.; Cartwright A. N.; Ilavsky J.; Rzayev J. Ultrahigh Molecular Weight Linear Block Copolymers: Rapid Access by Reversible-Deactivation Radical Polymerization and Self-Assembly into Large Domain Nanostructures. Macromolecules 2016, 49 (10), 3733–3738. 10.1021/acs.macromol.6b00863.

[ref14] Takano K.; Nyu T.; Maekawa T.; Seki T.; Nakatani R.; Komamura T.; Hayakawa T.; Hayashi T. Real-time and in situ observation of structural evolution of giant block copolymer thin film under solvent vapor annealing by atomic force microscopy. RSC Adv. 2020, 10 (1), 70–75. 10.1039/C9RA09043F. PMC9047986 35492547

[ref15] Kim E.; Ahn H.; Park S.; Lee H.; Lee M.; Lee S.; Kim T.; Kwak E.-A.; Lee J. H.; Lei X.; Huh J.; Bang J.; Lee B.; Ryu D. Y. Directed assembly of high molecular weight block copolymers: highly ordered line patterns of perpendicularly oriented lamellae with large periods. ACS Nano 2013, 7 (3), 1952–1960. 10.1021/nn3051264. 23441640

[ref16] Cummins C.; Alvarez-Fernandez A.; Bentaleb A.; Hadziioannou G.; Ponsinet V.; Fleury G. Strategy for Enhancing Ultrahigh-Molecular-Weight Block Copolymer Chain Mobility to Access Large Period Sizes (100 nm). Langmuir 2020, 36 (46), 13872–13880. 10.1021/acs.langmuir.0c02261. 33175555

[ref17] Hnatchuk N.; Hathaway E.; Cui J.; Li X. Nonequilibrium Self-Assembly of Ultrahigh-Molecular-Weight Block Copolymers into an Asymmetric Nanostructure. ACS Appl. Polym. Mater. 2022, 4 (10), 7311–7320. 10.1021/acsapm.2c01128.

[ref18] Appold M.; Gallei M. Bio-Inspired Structural Colors Based on Linear Ultrahigh Molecular Weight Block Copolymers. ACS Appl. Polym. Mater. 2019, 1 (2), 239–250. 10.1021/acsapm.8b00169.

[ref19] Cao W.; Xia S.; Jiang X.; Appold M.; Opel M.; Plank M.; Schaffrinna R.; Kreuzer L. P.; Yin S.; Gallei M.; Schwartzkopf M.; Roth S. V.; Müller-Buschbaum P. Self-Assembly of Large Magnetic Nanoparticles in Ultrahigh Molecular Weight Linear Diblock Copolymer Films. ACS Appl. Mater. Interfaces 2020, 12 (6), 7557–7564. 10.1021/acsami.9b20905. 31967448

[ref20] Hoheisel T. N.; Hur K.; Wiesner U. B. Block copolymer-nanoparticle hybrid self-assembly. Prog. Polym. Sci. 2015, 40, 3–32. 10.1016/j.progpolymsci.2014.10.002.

[ref21] Pryamitsyn V.; Ganesan V. Strong Segregation Theory of Block Copolymer–Nanoparticle Composites. Macromolecules 2006, 39 (24), 8499–8510. 10.1021/ma0613382.

[ref22] Sarkar B.; Alexandridis P. Block copolymer–nanoparticle composites: Structure, functional properties, and processing. Prog. Polym. Sci. 2015, 40, 33–62. 10.1016/j.progpolymsci.2014.10.009.

[ref23] Kim B. J.; Bang J.; Hawker C. J.; Chiu J. J.; Pine D. J.; Jang S. G.; Yang S.-M.; Kramer E. J. Creating surfactant nanoparticles for block copolymer composites through surface chemistry. Langmuir 2007, 23 (25), 12693–12703. 10.1021/la701906n. 17973409

[ref24] Gai Y.; Lin Y.; Song D.-P.; Yavitt B. M.; Watkins J. J. Strong Ligand–Block Copolymer Interactions for Incorporation of Relatively Large Nanoparticles in Ordered Composites. Macromolecules 2016, 49 (9), 3352–3360. 10.1021/acs.macromol.5b02609.

[ref25] Li Q.; Kartikowati C. W.; Horie S.; Ogi T.; Iwaki T.; Okuyama K. Correlation between particle size/domain structure and magnetic properties of highly crystalline Fe3O4 nanoparticles. Sci. Rep. 2017, 7 (1), 9894 10.1038/s41598-017-09897-5. 28855564 PMC5577113

[ref26] Patsula V.; Moskvin M.; Dutz S.; Horák D. Size-dependent magnetic properties of iron oxide nanoparticles. J. Phys. Chem. Solids 2016, 88, 24–30. 10.1016/j.jpcs.2015.09.008.

[ref27] Iida H.; Takayanagi K.; Nakanishi T.; Osaka T. Synthesis of Fe3O4 nanoparticles with various sizes and magnetic properties by controlled hydrolysis. J. Colloid Interface Sci. 2007, 314 (1), 274–280. 10.1016/j.jcis.2007.05.047. 17568605

[ref28] Xiao W.; Song W.; Herng T. S.; Qin Q.; Yang Y.; Zheng M.; Hong X.; Feng Y. P.; Ding J. Novel room-temperature spin-valve-like magnetoresistance in magnetically coupled nano-column Fe3O4/Ni heterostructure. Nanoscale 2016, 8 (34), 15737–15743. 10.1039/C6NR04805F. 27526860

[ref29] Wang H.; Jiao X.; Chen D. Monodispersed Nickel Nanoparticles with Tunable Phase and Size: Synthesis, Characterization, and Magnetic Properties. J. Phys. Chem. C 2008, 112 (48), 18793–18797. 10.1021/jp805591y.

[ref30] Chen Y.; Peng D.-L.; Lin D.; Luo X. Preparation and magnetic properties of nickel nanoparticles via the thermal decomposition of nickel organometallic precursor in alkylamines. Nanotechnology 2007, 18 (50), 505703 10.1088/0957-4484/18/50/505703.

[ref31] He X.; Zhong W.; Au C.-T.; Du Y. Size dependence of the magnetic properties of Ni nanoparticles prepared by thermal decomposition method. Nanoscale Res. Lett. 2013, 8 (1), 446 10.1186/1556-276X-8-446. 24164907 PMC4231360

[ref32] LaGrow A. P.; Ingham B.; Cheong S.; Williams G. V. M.; Dotzler C.; Toney M. F.; Jefferson D. A.; Corbos E. C.; Bishop P. T.; Cookson J.; Tilley R. D. Synthesis, alignment, and magnetic properties of monodisperse nickel nanocubes. J. Am. Chem. Soc. 2012, 134 (2), 855–858. 10.1021/ja210209r. 22239232

[ref33] Xia S.; Song L.; Hohn N.; Wang K.; Grott S.; Opel M.; Schwartzkopf M.; Roth S. V.; Müller-Buschbaum P. Spray-Coating Magnetic Thin Hybrid Films of PS- b -PNIPAM and Magnetite Nanoparticles. Adv. Funct. Mater. 2019, 29 (15), 1808427 10.1002/adfm.201808427.

[ref34] Hartmann F.; Bitsch M.; Niebuur B.-J.; Koch M.; Kraus T.; Dietz C.; Stark R. W.; Everett C. R.; Müller-Buschbaum P.; Janka O.; Gallei M. Self-Assembly of Polymer-Modified FePt Magnetic Nanoparticles and Block Copolymers. Materials 2023, 16 (16), 5503 10.3390/ma16165503. 37629794 PMC10455748

[ref35] Konefał M.; Černoch P.; Patsula V.; Pavlova E.; Dybal J.; Załęski K.; Zhigunov A. Enhanced Ordering of Block Copolymer Thin Films upon Addition of Magnetic Nanoparticles. ACS Appl. Mater. Interfaces 2021, 13 (7), 9195–9205. 10.1021/acsami.0c21549. 33565869

[ref36] Cano L.; Di Mauro A. E.; Petronella F.; Fanizza E.; Striccoli M.; Curri M. L.; Tercjak A. Effect of Iron Oxide Nanocrystal Content on the Morphology and Magnetic Properties of Polystyrene- block -poly(methyl methacrylate) Diblock Copolymer Based Nanocomposites. J. Phys. Chem. C 2015, 119 (11), 6435–6445. 10.1021/acs.jpcc.5b00634.

[ref37] Barandiaran I.; Grana E.; Katsigiannopoulos D.; Avgeropoulos A.; Kortaberria G. Nanocomposites based on nanostructured PI-b-PMMA copolymer and selectively placed PMMA-modified magnetic nanoparticles: Morphological and magnetic characterization. Eur. Polym. J. 2016, 75, 514–524. 10.1016/j.eurpolymj.2016.01.005.

[ref38] Komiyama H.; Hojo D.; Suzuki K. Z.; Mizukami S.; Adschiri T.; Yabu H. Binary Nanoparticles Coassembly in Bioinspired Block Copolymer Films: A Stepwise Synthesis Approach Using Multifunctional Catechol Groups and Magneto-Optical Properties. ACS Appl. Nano Mater. 2018, 1 (4), 1666–1674. 10.1021/acsanm.8b00141.

[ref39] Son J. G.; Bae W. K.; Kang H.; Nealey P. F.; Char K. Placement control of nanomaterial arrays on the surface-reconstructed block copolymer thin films. ACS Nano 2009, 3 (12), 3927–3934. 10.1021/nn900914q. 19916550

[ref40] Acharya H.; Sung J.; Sohn B.-H.; Kim D. H.; Tamada K.; Park C. Tunable Surface Plasmon Band of Position Selective Ag and Au Nanoparticles in Thin Block Copolymer Micelle Films. Chem. Mater. 2009, 21 (18), 4248–4255. 10.1021/cm901245g.

[ref41] Bockstaller M. R.; Lapetnikov Y.; Margel S.; Thomas E. L. Size-selective organization of enthalpic compatibilized nanocrystals in ternary block copolymer/particle mixtures. J. Am. Chem. Soc. 2003, 125 (18), 5276–5277. 10.1021/ja034523t. 12720430

[ref42] Horechyy A.; Nandan B.; Zafeiropoulos N. E.; Formanek P.; Oertel U.; Bigall N. C.; Eychmüller A.; Stamm M. A Step-Wise Approach for Dual Nanoparticle Patterning via Block Copolymer Self-Assembly. Adv. Funct. Mater. 2013, 23 (4), 483–490. 10.1002/adfm.201201452.

[ref43] Cao W.; Yin S.; Plank M.; Chumakov A.; Opel M.; Chen W.; Kreuzer L. P.; Heger J. E.; Gallei M.; Brett C. J.; Schwartzkopf M.; Eliseev A. A.; Anokhin E. O.; Trusov L. A.; Roth S. V.; Müller-Buschbaum P. Spray-Deposited Anisotropic Ferromagnetic Hybrid Polymer Films of PS-b-PMMA and Strontium Hexaferrite Magnetic Nanoplatelets. ACS Appl. Mater. Interfaces 2021, 13 (1), 1592–1602. 10.1021/acsami.0c19595. 33355441

[ref44] Chopra N.; Claypoole L.; Bachas L. G. Morphological control of Ni/NiO core/shell nanoparticles and production of hollow NiO nanostructures. J. Nanopart. Res. 2010, 12 (8), 2883–2893. 10.1007/s11051-010-9879-4.

[ref45] Reus M. A.; Baier T.; Lindenmeir C. G.; Weinzierl A. F.; Buyan-Arivjikh A.; Wegener S. A.; Kosbahn D. P.; Reb L. K.; Rubeck J.; Schwartzkopf M.; Roth S. V.; Müller-Buschbaum P. Modular slot-die coater for in situ grazing-incidence x-ray scattering experiments on thin films. Rev. Sci. Instrum. 2024, 95 (4), 043907 10.1063/5.0204673. 38656556

[ref46] Buffet A.; Rothkirch A.; Döhrmann R.; Körstgens V.; Abul Kashem M. M.; Perlich J.; Herzog G.; Schwartzkopf M.; Gehrke R.; Müller-Buschbaum P.; Roth S. V. P03, the microfocus and nanofocus X-ray scattering (MiNaXS) beamline of the PETRA III storage ring: the microfocus endstation. J. Synchrotron Radiat. 2012, 19 (4), 647–653. 10.1107/S0909049512016895. 22713902 PMC3380660

[ref47] Sing C. E.; Zwanikken J. W.; La Olvera de Cruz M. Electrostatic control of block copolymer morphology. Nat. Mater. 2014, 13 (7), 694–698. 10.1038/nmat4001. 24907928

[ref48] Wang X.; Goswami M.; Kumar R. G.; Sumpter B. G.; Mays J. Morphologies of block copolymers composed of charged and neutral blocks. Soft Matter 2012, 8 (11), 3036 10.1039/c2sm07223h.

[ref49] Ham S.; Shin C.; Kim E.; Ryu D. Y.; Jeong U.; Russell T. P.; Hawker C. J. Microdomain Orientation of PS-b-PMMA by Controlled Interfacial Interactions. Macromolecules 2008, 41 (17), 6431–6437. 10.1021/ma8007338.

[ref50] Leclère P.; Lazzaroni R.; Brédas J. L.; Yu J. M.; Dubois P.; Jérôme R. Microdomain Morphology Analysis of Block Copolymers by Atomic Force Microscopy with Phase Detection Imaging. Langmuir 1996, 12 (12), 4317–4320. 10.1021/la9600964.

[ref51] Li Y.; Huang H.; He T.; Gong Y. The effect of the preferential affinity of the solvent on the microstructure of solution-cast block copolymer thin films. J. Phys. Chem. B 2010, 114 (3), 1264–1270. 10.1021/jp908852u. 20041725

[ref52] Posselt D.; Zhang J.; Smilgies D.-M.; Berezkin A. V.; Potemkin I. I.; Papadakis C. M. Restructuring in block copolymer thin films: In situ GISAXS investigations during solvent vapor annealing. Prog. Polym. Sci. 2017, 66, 80–115. 10.1016/j.progpolymsci.2016.09.009.

[ref53] Brassat K.; Lindner J. K. N. Nanoscale Block Copolymer Self-Assembly and Microscale Polymer Film Dewetting: Progress in Understanding the Role of Interfacial Energies in the Formation of Hierarchical Nanostructures. Adv. Mater. Interfaces 2020, 7 (5), 1901565 10.1002/admi.201901565.

[ref54] Ul-Hamid A. Contrast Formation in the SEM. In A Beginners’ Guide to Scanning Electron Microscopy; Springer International Publishing, 2018; . 10.1007/978-3-319-98482-7.

[ref55] Kucuk N.; Cakir M.; Isitman N. A. Mass attenuation coefficients, effective atomic numbers and effective electron densities for some polymers. Radiat. Prot. Dosim. 2013, 153 (1), 127–134. 10.1093/rpd/ncs091. 22645382

[ref56] Müller-Buschbaum P. Grazing incidence small-angle X-ray scattering: an advanced scattering technique for the investigation of nanostructured polymer films. Anal. Bioanal. Chem. 2003, 376 (1), 3–10. 10.1007/s00216-003-1869-2. 12734612

[ref57] Müller-Buschbaum P. A. Basic Introduction to Grazing Incidence Small-Angle X-Ray Scattering. In Applications of Synchrotron Light to Scattering and Diffraction in Materials and Life Sciences; Springer: Berlin Heidelberg, 2009; pp 61–89. 10.1007/978-3-540-95968-7.

[ref58] Sinha S. K.; Sirota E. B.; Garoff S.; Stanley H. B. X-ray and neutron scattering from rough surfaces. Phys. Rev. B 1988, 38, 2297 10.1103/PhysRevB.38.2297. 9946532

[ref59] Holý V.; Kuběna J.; Ohlídal I.; Lischka K.; Plotz W. X-ray reflection from rough layered systems. Phys. Rev. B 1993, 47, 15896–15903. 10.1103/PhysRevB.47.15896. 10005989

[ref60] Gu Y.; Dorin R. M.; Tan K. W.; Smilgies D.-M.; Wiesner U. In Situ Study of Evaporation-Induced Surface Structure Evolution in Asymmetric Triblock Terpolymer Membranes. Macromolecules 2016, 49 (11), 4195–4201. 10.1021/acs.macromol.6b00265.

[ref61] Seguini G.; Zanenga F.; Giammaria T. J.; Ceresoli M.; Sparnacci K.; Antonioli D.; Gianotti V.; Laus M.; Perego M. Enhanced Lateral Ordering in Cylinder Forming PS-b-PMMA Block Copolymers Exploiting the Entrapped Solvent. ACS Appl. Mater. Interfaces 2016, 8 (12), 8280–8288. 10.1021/acsami.6b00360. 26959626

[ref62] Opel M.; Geprägs S.; Menzel E. P.; Nielsen A.; Reisinger D.; Nielsen K.-W.; Brandlmaier A.; Czeschka F. D.; Althammer M.; Weiler M.; Goennenwein S. T. B.; Simon J.; Svete M.; Yu W.; Hühne S.-M.; Mader W.; Gross R. Novel multifunctional materials based on oxide thin films and artificial heteroepitaxial multilayers. Phys. Status Solidi A 2011, 208 (2), 232–251. 10.1002/pssa.201026403.

[ref63] Binek C.; Polisetty S.; He X.; Berger A. Exchange bias training effect in coupled all ferromagnetic bilayer structures. Phys. Rev. Lett. 2006, 96 (6), 67201 10.1103/PhysRevLett.96.067201. 16606037

[ref64] Daffe N.; Zecevic J.; Trohidou K. N.; Sikora M.; Rovezzi M.; Carvallo C.; Vasilakaki M.; Neveu S.; Meeldijk J. D.; Bouldi N.; Gavrilov V.; Guyodo Y.; Choueikani F.; Dupuis V.; Taverna D.; Sainctavit P.; Juhin A. Bad neighbour, good neighbour: how magnetic dipole interactions between soft and hard ferrimagnetic nanoparticles affect macroscopic magnetic properties in ferrofluids. Nanoscale 2020, 12, 11222–11231. 10.1039/d0nr02023k. 32412032

[ref65] Omelyanchik A.; Villa S.; Locardi F.; Ferretti A. M.; Ponti A.; Singh G.; Barucca G.; Slimami S.; Tedesco A. D.; Riello P.; Rutkowski B.; Vasilakaki M.; Margaris G.; Trohidou K. N.; Peddis D. Magnetic Anisotropy and Interactions in Hard/Soft Core/Shell Nanoarchitectures: The Role of Shell Thickness. Chem. Mater. 2024, 36, 7976–7987. 10.1021/acs.chemmater.4c01421.

[ref66] Vamvakidis K.; Mourdikoudis S.; Makridis A.; Paulidou E.; Angelakeris M.; Dendrinou-Samara C. Magnetic hyperthermia efficiency and MRI contrast sensitivity of colloidal soft/hard ferrite nanoclusters. J. Colloid Interface Sci. 2018, 511, 101–109. 10.1016/j.jcis.2017.10.001. 28992447

[ref67] Brown W. F. Jr. Thermal Fluctuations of a Single-Domain Particle. Phys. Rev. 1963, 130, 1677–1686. 10.1103/PhysRev.130.1677.

[ref68] Baker C.; Ismat Shah S.; Hasanain S. Magnetic behavior of iron and iron-oxide nanoparticle/polymer composites. J. Magn. Magn. Mater. 2004, 280 (2–3), 412–418. 10.1016/j.jmmm.2004.03.037.

[ref69] Carvalho M. D.; Henriques F.; Ferreira L. P.; Godinho M.; Cruz M. M. Iron oxide nanoparticles: the Influence of synthesis method and size on composition and magnetic properties. J. Solid State Chem. 2013, 201, 144–152. 10.1016/j.jssc.2013.02.024.

