# Supplementary material for: Printed Thin Magnetic Films via Ternary Hybrid Diblock Copolymer Films Containing Magnetic Iron Oxide and Nickel Nanoparticles
Source: ACS Appl Mater Interfaces. 2024 Dec 11;16(51):71060–9. doi: 10.1021/acsami.4c18920 (PMC11672476; doi:10.1021/acsami.4c18920)
Supplement: Supplementary file 1 — am4c18920_si_001.pdf [file am4c18920_si_001.pdf]

Supporting Information

Printed thin magnetic films via ternary hybrid diblock copolymer films containing magnetic iron oxide and nickel nanoparticles

Christopher R. Everett[1], Xinyu Jiang[2], Manuel A. Reus[1], Huaying Zhong[1], Martin Bitsch[3], Martina Plank[4], Markus Gallei[3,5], Matthias Opel[6], Matthias Schwartzkopf[2], Stephan V. Roth[2,7], Peter Müller-Buschbaum[1,]*

[1] Technical University of Munich, TUM School of Natural Sciences, Department of Physics, Chair for Functional Materials, James-Franck-Str. 1, 85748 Garching, Germany

[2] Deutsches Elektronen-Synchrotron (DESY), Notkestraße 85, 22607 Hamburg, Germany

[3] Polymer Chemistry, Saarland University, Campus C4 2, 66123 Saarbrücken, Germany

[4] Ernst-Berl-Institute for Technical and Macromolecular Chemistry, Technische Universität Darmstadt, Alarich-Weiss-Straße 4, 64287 Darmstadt, Germany

[5] Saarene, Saarland Center for Energy Materials and Sustainability, Campus C4 2, 66123 Saarbrücken, Germany

[6] Walther-Meissner-Institut, Bayerische Akademie der Wissenschaften, , Walther-Meissner-Straße 8, 85748 Garching, Germany

[7] Department of Fibre and Polymer Technology, KTH Royal Institute of Technology, Teknikringen 56-58, SE-100 44 Stockholm, Sweden

[*]Email: muellerb@ph.tum.de

Synthesis of Ni NPs:

Nickel NPs (Ni, d_{TEM} = 46 ± 10 nm) were synthesized according to Nitin Chopra et al. 0.99 g Nickel(II) acetate tetrahydrate and 7 mL oleylamine were added to a 100 mL three-necked round bottom flask equipped with a mechanic stirrer under inert gas atmosphere. In the next step the reaction was heated up to 80 °C in a metal bath for 30 min. Then 3.43 mL tri-*n*-octylphosphine oxide (TOPO) and 1 mL tri-*n*-octylphosphine (TOP) were added to the flask, and the mixture was heated up to 250 °C in a metal bath for 2 h. After cooling down to room temperature, the particles were washed several times with ethanol.

Modification of Ni NPs with 2-bromo-2-methyl-*N*-(3-(triethoxysilyl)propyl) propenamide

55 mg of the synthesized Ni NPs were dispersed in 25 mL toluene and added to a round bottom Schlenk flask equipped with a mechanical stirrer under an argon atmosphere. Subsequently, 0.1 mL triethylamine and 0.2 mL 2-bromo-2-methyl-*N*-(3-(triethoxysilyl)propyl) propenamide were added, and the mixture was stirred for 48 h at room temperature. Then the particles were centrifuged and washed two times with toluene and two times with anisole. Afterwards, the modified Ni NPs were dispersed in 25 mL anisole.

Surface functionalization with poly(methyl methacrylate) (PMMA)

25 mL of the modified Ni NPs dispersion were added to a three-necked round bottom Schlenk flask equipped with a mechanical stirrer and an argon inlet. After the addition of 10 mL methyl methacrylate (MMA) and 3 µL *tert*-butyl-α-bromisobutyrate, the dispersion was heated up to 90 °C. The polymerization was initiated by the addition of 2 mL of a [Cu^{I}(PMDETA)Cl] solution (2 M in anisole). After 4 h, the mixture was precipitated in an excess of methanol and separated by filtration. The precipitate was redispersed in THF, separated by centrifugation, and washed several times with THF.

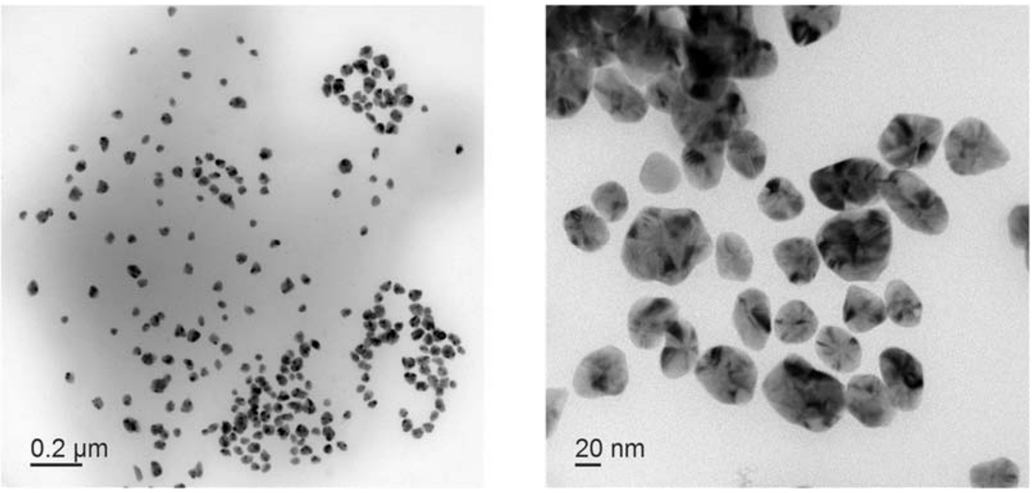

Figure S1: TEM images of the synthesized Ni NPs after surface functionalization with PMMA.

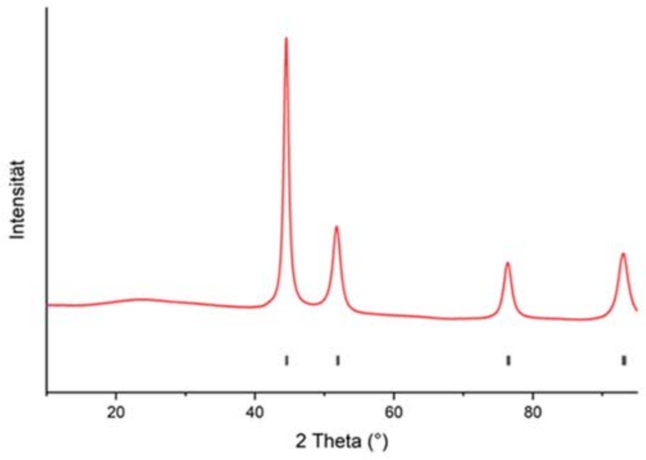

Figure S2: XRD diffraction pattern of the as-prepared Ni NPs (red curve). Ni peaks corresponding to a face-centered cubic structure are marked with grey lines (JCPDS card no. 04-0850).

Acid solution cleaning procedure of Si substrates:

Pre-cut Si substrates were submersed for 15 minutes into a solution of 54 mL deionized water (DI H_2O), 84 mL H_2O_2, and 198 mL H_2SO_4 at 80°C. After the acid bath, the substrates were rinsed again with DI H_2O and dried with nitrogen gas

Table S1: Thicknesses of the hybrid films prepared during the in situ printing investigation as measured by spectral reflectance. A Filmetrics F20 Thin-Film Analyzer (KLA) with a spot size of 2.0 mm was utilized, and the thickness was determined by fits to the reflected spectrum with the FILMeasure software (KLA) robust fit algorithm. Measurements were taken at four sample positions and averaged together.

Sample	thickness [nm]
No NPs	314 ± 3
1 wt % Fe_3O_4 and 1 wt % Ni	320 ± 7
1 wt % Fe_3O_4 and 2 wt % Ni	276 ± 9
1 wt % Fe_3O_4 and 5 wt % Ni	305 ± 2

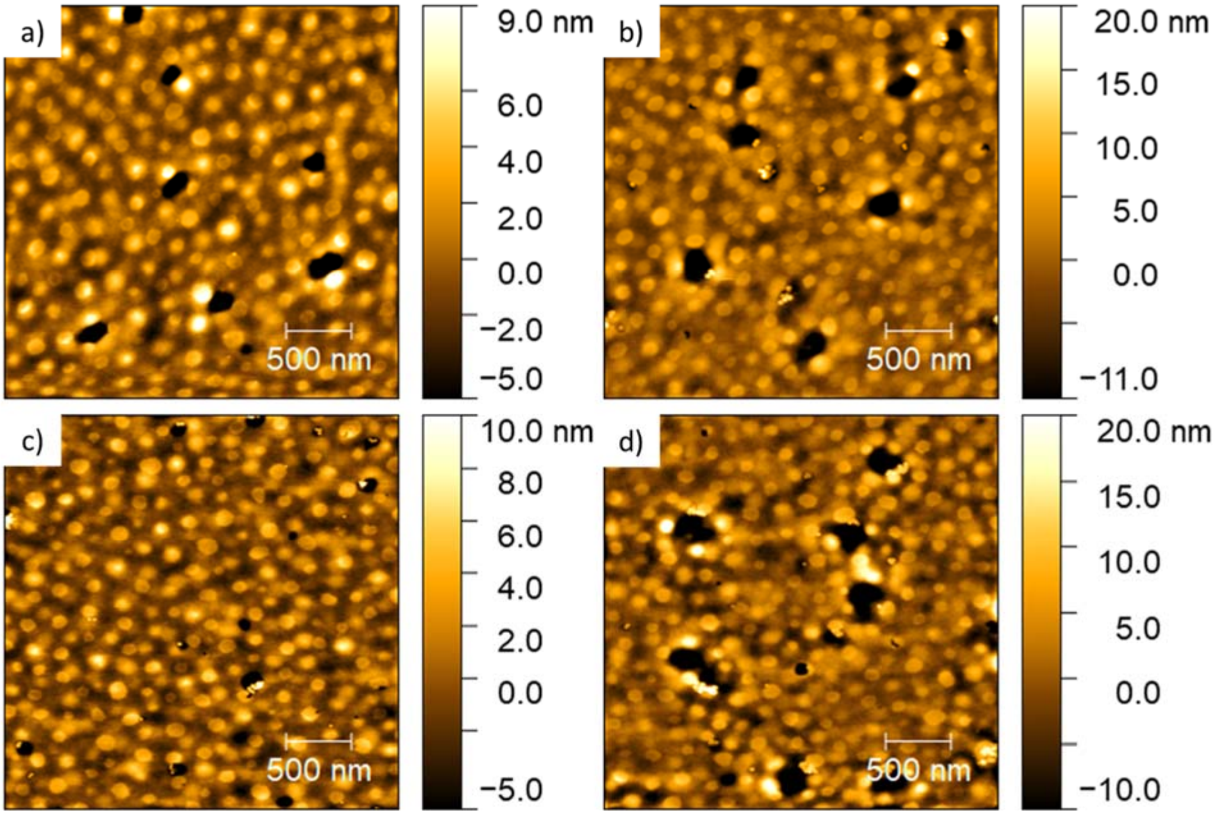

Figure S3. AFM topography images of ternary hybrid films printed during the in situ characterization with increasing NP concentration: (a) no NPs, (b) 1 wt % Fe_3O_4 and 1 wt % Ni, (c) 1 wt % Fe_3O_4 and 2 wt % Ni, (d) 1 wt % Fe_3O_4 and 5 wt % Ni. NPs can be primarily observed as agglomerates.

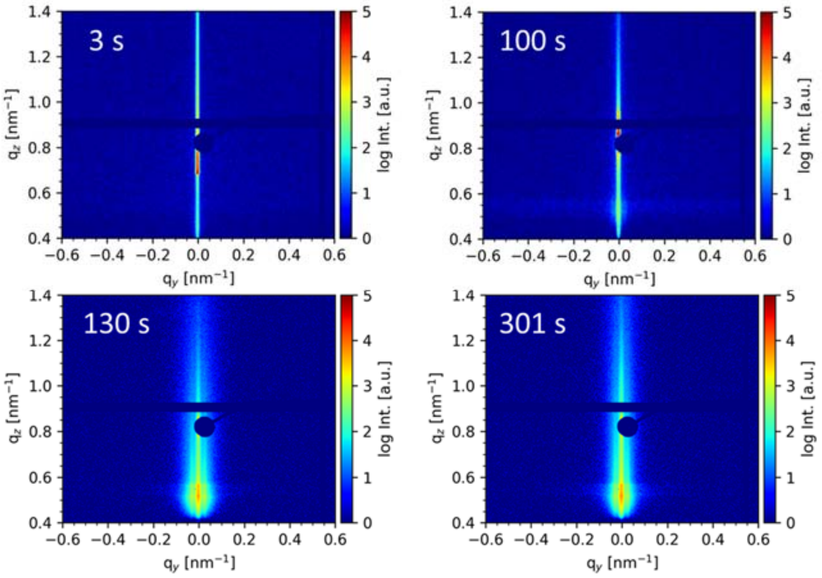

Figure S4. Selected 2D GISAXS data during the printing process for the PS-*b*-PMMA film with no NPs. The time at which each image is collected is shown in the top right of each image. The specular beam is covered by a round beam stop to avoid over-saturation of the detector. The evolution of the film morphology out of the wet film state can be observed.

Modeling of GISAXS data:

For the quantitative analysis of the lateral structures inside the DBC films, horizontal line cuts of the 2D GISAXS data (shown in Figure S4 and Figure S6) were taken at the Yoneda region of the PS-*b*-PMMA films using the software DPDAK.[1] The line cuts were then modeled within the scope of the distorted-wave Born approximation (DWBA) and the local monodisperse approximation (LMA).[2] Each scattering component is described by a form factor, related to the shape of the object, and a structure factor, related to the distribution of the objects in relation to one another. The scattering intensity from one scattering object can be written as Equation S1:

$$I(q) \propto N \cdot |F(q)|^2 \cdot S(q)$$

with N being the number of scattering objects, $F(q)$ being the form factor, and $S(q)$ being the structure factor. The total scattering intensity is then the sum of the scattering intensities of the distinct scattering object within the film. In this study, the polymer domains are modeled with a singular cylindrical form factor with Gaussian size distribution in the mean radius, R_i, and a center-to-center distance between the cylindrical structures with the mean value, D_i. The cylindrical form factor is approximated as

$$\left|F_{cyl}(q_y)\right|^2 \approx \left(R\frac{J_1(q_y, R)}{q_y}\right)^2$$

where J_1 is the Bessel function of the first kind, q_y is the momentum transfer in the horizontal direction, and R is the domain radius. The NPs are modeled similarly with spherical form factors and are also described by a structure factor. More information on the form factors can be found elsewhere.[3] Using the Hosemann interference function, the respective structure factors model the center-to-center distances of the scattering object on a 1D paracrystalline lattice[4]:

$$S(q_y) = -\frac{1 - \exp\left(\pi\sigma_D^2 D^2 q_y^2\right)^2}{1 + \exp\left(\pi\sigma_D^2 D^2 q_y^2\right)^2 - 2\exp\left(\pi\sigma_D^2 D^2 q_y^2\right)\cos\left(q_y D\right)}$$

Here σ_D is the Gaussian standard deviation of the mean value of the center-to-center distance, D is the mean center-to-center distance, and q_y is the horizontal component of the momentum transfer. The mean object sizes and distances are obtained from the fits to the data.

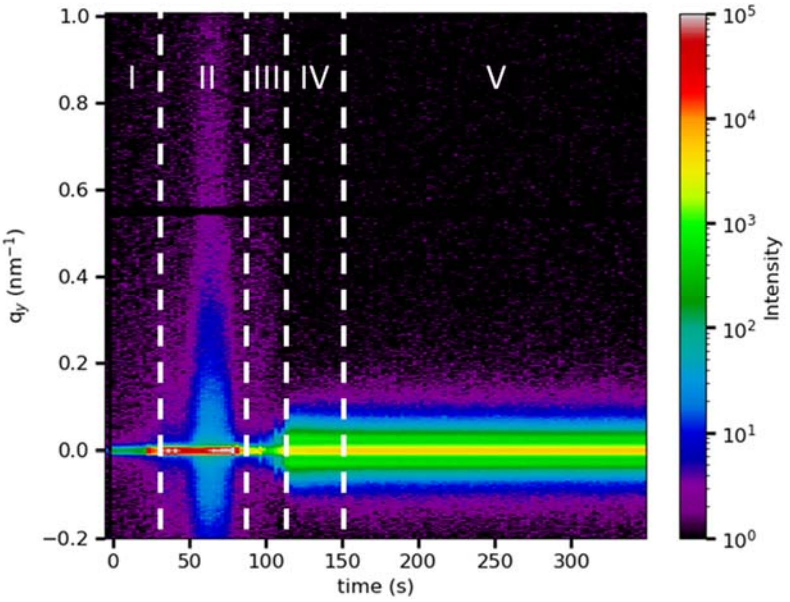

Figure S5. 2D color map of horizontal line cuts collected during the in situ slot-die printing process of the PS-*b*-PMMA film with no NPs. The five stages of film formation described in the main text are shown.

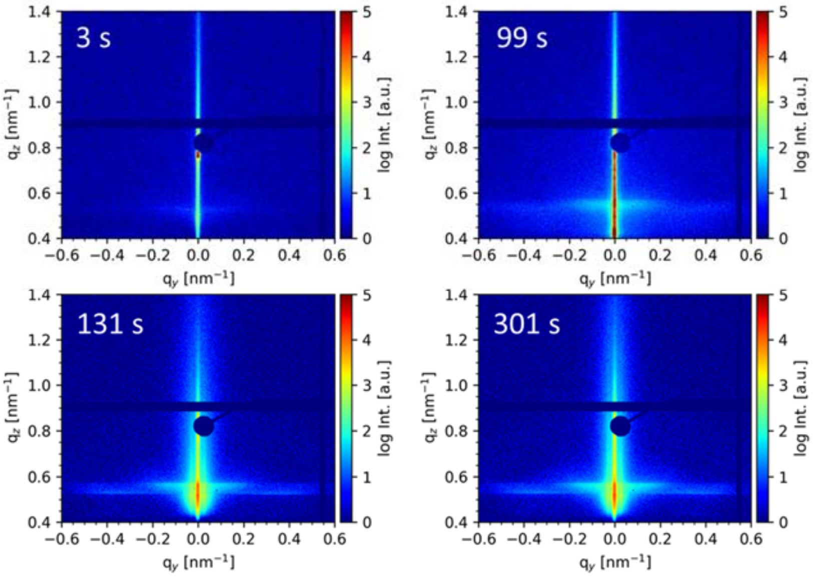

Figure S6. Selected 2D GISAXS data during the printing process for the PS-*b*-PMMA film with 1 wt % Fe_3O_4 NPs and 1 wt % Ni NPs. The time at which each image is collected is shown in the top right of each image. The specular beam is covered by a round beam stop to avoid over-saturation of the detector. The evolution of the film morphology out of the wet film state can be observed.

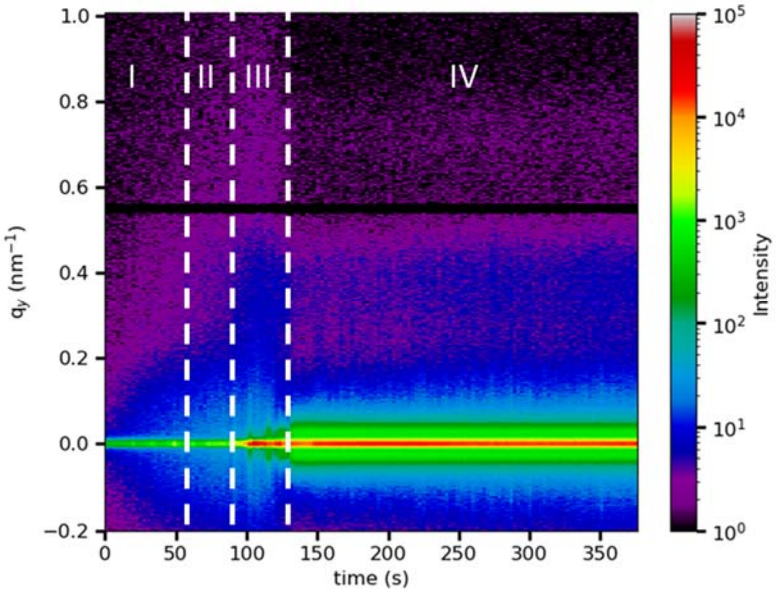

Figure S7. 2D color map of each horizontal line cut collected during the in situ slot-die printing process of the PS-*b*-PMMA film with 1 wt % Fe_3O_4 NPs and 1 wt % Ni NPs. The four stages of film formation described in the main text are shown.

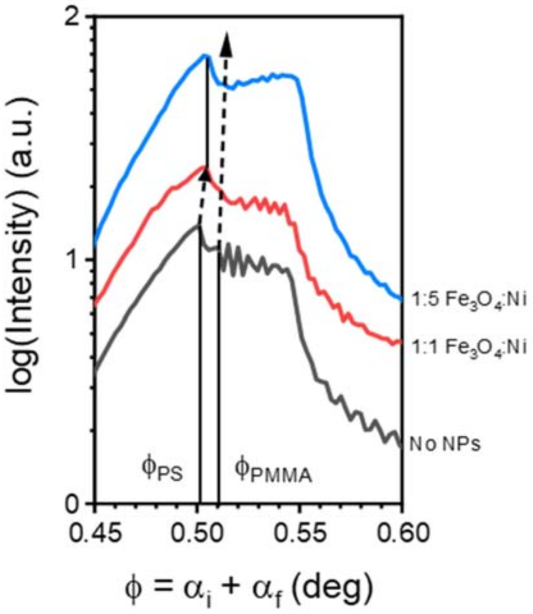

Figure S8. Vertical line cuts of the 2D GISAXS data of the as-prepared films printed during the in situ characterization with different concentrations of NPs. For clarity, the cuts are shifted along the y-axis. The in-plane scattering angle, φ, is calculated as the sum of the incidence angle, α_i, and the exit angle, α_f. The Yoneda peaks of PS and PMMA appear at the in-plane scattering angles where α_f equals the respective critical angle α_c. Upon the addition of NPs, the critical angles of PS and PMMA increase, as shown by the dashed lines. Further increase of the concentration of Ni NPs correlates to a further increase in the critical angle of PMMA.

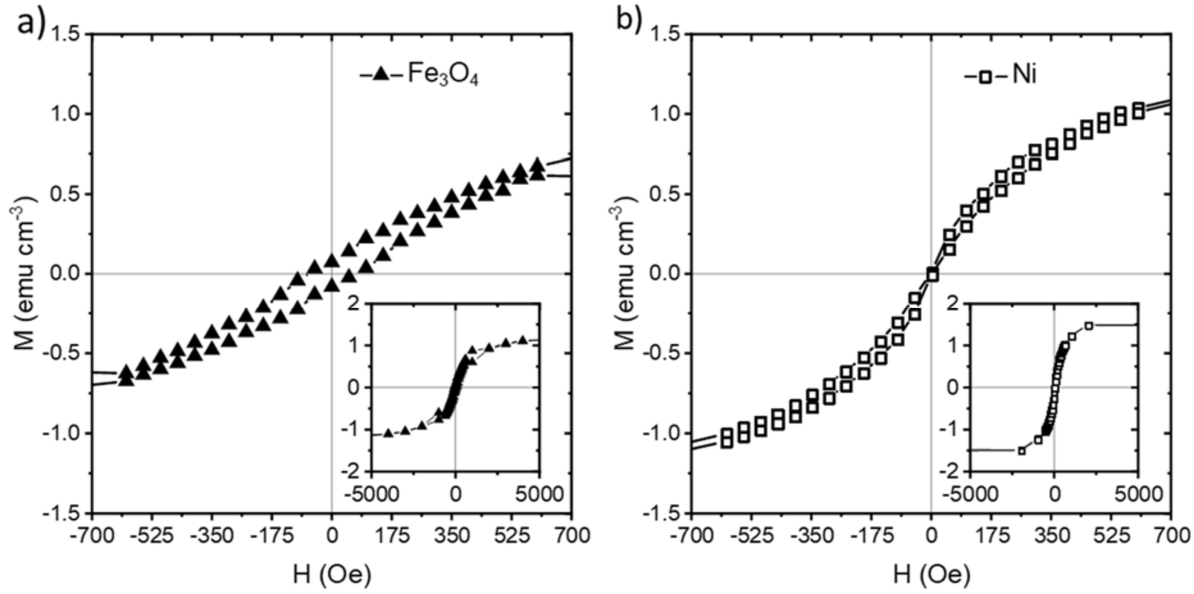

Figure S9. Magnetic hysteresis curves plotted between -700 Oe and 700 Oe for binary hybrid DBC films containing (a) 2 wt % Fe_3O_4 NPs and (b) 2wt % Ni NPs. The insets show each curve between -5000 Oe and 5000 Oe. The films were prepared in the same manner as the ternary hybrid films. For the binary film with 2 wt % Fe_3O_4 NPs, the saturation magnetization M_s, remanence M_r, and coercivity H_c are approximately 1.2 emu cm^{-3}, 0.08 emu cm^{-3}, and 71 Oe. For the film containing 2 wt % Ni NPs, M_s, M_r, and H_c are approximately 1.5 emu cm^{-3}, 0.01 emu cm^{-3}, and 2 Oe.

REFERENCES

[1] Benecke, G.; Wagermaier, W.; Li, C.; Schwartzkopf, M.; Flucke, G.; Hoerth, R.; Zizak, I.; Burghammer, M.; Metwalli, E.; Müller-Buschbaum, P.; Trebbin, M.; Förster, S.; Paris, O.; Roth, S. V.; Fratzl, P. A Customizable Software for Fast Reduction and Analysis of Large X-Ray Scattering Data Sets: Applications of the New DPDAK Package to Small-Angle X-Ray Scattering and Grazing-Incidence Small-Angle X-Ray Scattering. *J. Appl. Crystallogr.* **2014**, 47 (5), 1797–1803. DOI: 10.1107/S1600576714019773.

[2] Hexemer, A.; Müller-Buschbaum, P. Advanced Grazing-Incidence Techniques for Modern Soft-Matter Materials Analysis. *IUCrJ* **2015**, 2 (1), 106–125. DOI: 10.1107/S2052252514024178

[3] R. Lazzari, ”IsGISAXS : A program for grazing-incidence small-angle X-ray scattering analysis of supported islands“, *J. Appl. Cryst*. **2002**, 34(4), 406–421. DOI: 10.1107/S0021889802006088.

[4] R. Hosemann, ”Crystalline and paracrystalline order in high polymers“, *J. Appl. Phys.* **1963**, 34(1) 25–41. DOI: 10.1063/1.1729085.
